# Draft Genome Sequences of *Gluconobacter cerinus* CECT 9110 and *Gluconobacter japonicus* CECT 8443, Acetic Acid Bacteria Isolated from Grape Must

**DOI:** 10.1128/genomeA.00621-16

**Published:** 2016-06-30

**Authors:** Florencia Sainz, Albert Mas, María Jesús Torija

**Affiliations:** Biotecnologia Enológica. Department Bioquímica i Biotecnologia, Facultat d’Enologia, Universitat Rovira i Virgili, Tarragona, Spain

## Abstract

We report here the draft genome sequences of *Gluconobacter cerinus* strain CECT9110 and *Gluconobacter japonicus* CECT8443, acetic acid bacteria isolated from grape must. *Gluconobacter* species are well known for their ability to oxidize sugar alcohols into the corresponding acids. Our objective was to select strains to oxidize effectively d-glucose.

## GENOME ANNOUNCEMENT

The species *Gluconobacter cerinus* (1) was reclassified to *G. cerinus* in 1984 (2) after protein electrophoretic profiles analysis. This species was studied due to the production of ketofructose (3) and some particularities in lipid composition (4, 5). Instead, the species *Gluconobacter japonicus* was proposed in 2009 after DNA hybridization within the genus *Gluconobacter* (6). The type strain had been isolated from the fruits of *Myrica rubra* in Japan. The genus *Gluconobacter* is included in acetic acid bacteria (AAB) and has preference for sugary substrates. AAB are Gram-negative bacteria from the family *Acetobacteraceae*. AAB are aerobic microorganisms that are the main bacteria responsible for vinegar production but also for other biotechnological applications (7). AAB produce the corresponding ketones, aldehydes, and acids from the incomplete oxidation of a wide range of carbohydrates and alcohols. The oxidized products can be recovered from media (8). The biotechnological industry has taken advantage of this capacity of *Gluconobacter* species to recover some compounds, such as intermediaries, in the production of vitamin C (l-sorbose) and miglitol (antidiabetic drug, after amino-l-sorbose) (9, 10).

Both species have been described in grapes or grape musts. *G. cerinus* was isolated in grape musts from Spain (11) and rotten grapes in Australia (12), whereas *G. japonicus* has been recovered in Spain, both in grape musts (11) as well as in fermented musts (13). *G. japonicus* was also detected in kefir grains by culture independent methods (denaturing gradient gel electrophoresis PCR [DGGE-PCR]) (14). The present strains were isolated in our experimental cellar in Tarragona, Spain (11).

Our interest in these strains was for their use in the production of gluconate from d-glucose without the oxidation of fructose (15). This transformation is sought for use in the production of new strawberry beverages based on the presence of fructose as a sweetener and free of glucose because it is converted into gluconic acid or the corresponding gluconates (16–18).

Genomic DNA was extracted according to the cetyltrimethylammonium bromide (CTAB) method (19). For whole-genome sequencing, the Genome Analyzer Ion Torrent PGM (Thermo Fisher Scientific, Madrid, Spain) was used. Preparation of shotgun libraries was performed according to the protocols of the manufacturers and resulted in 5,149,025 reads (256 bp).

The genomes consisted of a chromosome with 3.66 Mb and an overall G+C content of 55.68% for *G. cerinus*, and 3.50 Mb and a G+C content of 56.28% for *G. japonicus*. The genomes were assembled in 45 contigs from 1,529,910 reads (*G. cerinus*) and 50 contigs from 1,831,761 reads (*G. japonicus*) using the software MIRA 4.9.5_2 (20). Prokka (21) was used for automatic annotation and gene detection. The genome harbored 2 rRNA genes, 49 tRNA genes, 2,616 protein-coding genes with predicted functions, and 786 genes coding for hypothetical proteins for *G. cerinus*, and 2 rRNA genes, 53 tRNA genes, 2,594 protein-coding genes with predicted functions, and 645 genes coding for hypothetical proteins for *G. japonicus*.

Among the identified genes, 122 and 132 encoded dehydrogenases in *G. cerinus* and *G. japonicus*, respectively, including membrane PQQ-dependent glucose dehydrogenase, flavin adenine dinucleotide (FAD)-dependent gluconate-2-dehydrogenase, and PQQ-dependent sorbitol dehydrogenase. Both strains have two genes encoding PQQ-dependent sorbitol dehydrogenase.

### Nucleotide sequence accession numbers.

This whole-genome shotgun project of *G. cerinus* CECT 9110 and *G. japonicus* CECT 8443 has been deposited at DDBJ/EMBL/GenBank under the accession numbers LUTU00000000 and LVHE00000000, respectively. The versions described in this paper are versions LUTU01000000 for *G. cerinus* CECT 9110 and LVHE01000000 for *G. japonicus* CECT 8443.
